# Electrokinetic analysis reveals common conditioner ingredient interactions with human hair

**DOI:** 10.1111/ics.70038

**Published:** 2025-10-22

**Authors:** Huijun Phoebe Tham, Kah Yuen Yip, Thomas Luxbacher, Roger L. McMullen, Thomas L. Dawson

**Affiliations:** ^1^ A*STAR Skin Research Labs (A*SRL) Agency for Science, Technology and Research (A*STAR) Singapore City Singapore; ^2^ Anton Paar GmbH Graz Austria; ^3^ Ashland Specialty Ingredients, G.P. Bridgewater New Jersey USA; ^4^ Center for Cell Death, Injury Regeneration, Department of Drug Discovery Biomedical Sciences and Biochemistry Molecular Biology Medical University of South Carolina Charleston South Carolina USA; ^5^ A*STAR Skin Research Labs (A*SRL) Agency for Science, Technology and Research (A*STAR) & Skin Research Institute of Singapore (SRIS) Singapore City Singapore

**Keywords:** chemical analysis, delivery/vectorization/penetration, formulation/stability, hair treatment

## Abstract

**Objective:**

This study aims to use electrokinetic analysis to investigate the deposition behaviour and conditioning efficacy of cationic surfactants on human hair, focusing on how surfactant structure, concentration and hair damage influence performance. It also aims to understand the mechanisms governing surfactant adsorption and their impact on hair manageability and health.

**Methods:**

This research employed streaming potential measurements—including pH‐dependent, time‐dependent and concentration‐dependent zeta (ζ)‐potential studies—alongside wet combing analyses and ATR‐FTIR spectroscopy to evaluate the adsorption affinity and conditioning effects of four cationic surfactants: behentrimonium chloride (BTMC), behentrimonium methosulfate (BTMS), hexadecyltrimethylammonium chloride (CTAC) and stearylalkonium chloride (STAC). A simplified model surface using silicon oxide (Si | SiO_2_) wafers was also utilized to isolate the influence of hair's natural variability and fibrous structure.

**Results:**

Longer‐chain surfactants like BTMC showed superior deposition and conditioning due to stronger van der Waals interactions, while bulky groups in STAC hindered deposition. BTMC outperformed BTMS, likely due to the chloride counterion's higher mobility. BTMC and BTMS were superior against CTAC and STAC due to their longer carbon chain length. Wet combing analyses revealed that BTMC significantly reduced combing forces, improving manageability, whereas STAC fared the worst due to its low adsorption. However, ATR‐FTIR analysis indicated no reversal of oxidative damage, suggesting conditioners improve manageability without repairing structural damage.

**Conclusion:**

The study highlights the importance of surfactant molecular structure—such as carbon chain length and counterion type—in deposition efficiency and conditioning performance, providing valuable insights for developing more effective hair care formulations. By leveraging electrokinetic analyses in the form of streaming potential experiments, we were able to quantitatively assess adsorption behaviour, ζ‐potential changes and the dynamic interactions between surfactants and hair. These findings enhance the understanding of cationic surfactant‐hair interactions, offering practical implications for optimizing conditioners to improve user experience and hair health.

## Graphical abstract

Novel electrokinetic analysis reveals how cationic surfactant deposition on hair is influenced by chain length and counterion mobility. Longer‐chain surfactants show superior adsorption through enhanced van der Waals interactions, while chloride counterions enable stronger electrostatic interactions. Higher deposition correlates with reduced combing forces. This approach enables rational conditioning formulation design.

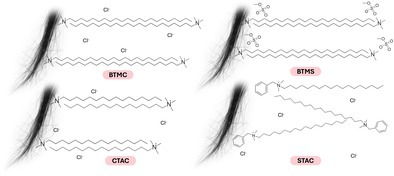

## Résumé

**Objectif:**

Cette étude vise à utiliser une analyse électrocinétique pour évaluer le comportement de dépôt et l'efficacité de conditionnement des tensioactifs cationiques sur les cheveux humains, en se concentrant sur la façon dont la structure et la concentration des tensioactifs et le degré de dommage capillaire influencent les performances. Elle vise également à comprendre les mécanismes qui régissent l'adsorption des tensioactifs et leur impact sur la facilité de coiffage et la santé des cheveux.

**Méthodes:**

Cette recherche a utilisé des mesures du potentiel de diffusion (études de potentiel zêta [ζ] en fonction du pH, du temps et de la concentration), ainsi que des analyses de peignage humide et une spectroscopie ATR‐FTIR pour évaluer l'affinité d'adsorption et les effets de conditionnement de quatre tensioactifs cationiques: chlorure de béhentrimonium (BTMC), méthosulfate de béhentrimonium (BTMS), chlorure d'hexadécyltriméthylammonium (CTAC) et chlorure de stéaralkonium (STAC). Un modèle de surface simplifié utilisant des plaquettes d'oxyde de silicium (Si |SiO_2_) a également été utilisé pour isoler l'influence de la variabilité naturelle des cheveux et de la structure de la fibre capillaire.

**Résultats:**

Les tensioactifs à longue chaîne, comme le BTMC, ont montré un dépôt et un conditionnement supérieurs en raison d'interactions de Van der Waals plus fortes, tandis que les groupes volumineux en STAC ont limité le dépôt. Le BTMC a surpassé le BTMS, probablement en raison de la plus grande mobilité du contre‐ion chlorure. Le BTMC et le BTMS se sont avérés supérieurs au CTAC et au STAC en raison d'une longueur de chaîne de carbone plus importante. Les analyses de peignage humide ont révélé que le BTMC réduisait significativement les forces de peignage et améliorait la facilité de coiffage, tandis que les plus mauvais résultats ont été obtenus avec le STAC en raison de son faible niveau d'absorption. Cependant, l'analyse par ATR‐FTIR n'a pas montré d'inversion des dommages oxydatifs, ce qui suggère que les après‐shampooings améliorent la facilité de coiffage sans réparer les dommages structurels.

**Conclusion:**

L'étude souligne l'importance de la structure moléculaire des tensioactifs, comme la longueur de la chaîne de carbone et le type de contre‐ion, dans l'efficacité de dépôt et les performances de conditionnement, fournissant ainsi des informations précieuses pour développer des formulations de soins capillaires plus efficaces. En utilisant des analyses électrocinétiques sous forme d'expériences de potentiel de diffusion, nous avons pu évaluer quantitativement le comportement d'absorption, les changements de potentiel ζ et les interactions dynamiques entre les tensioactifs et les cheveux. Ces résultats permettent de mieux comprendre les interactions entre les surfactants cationiques et les cheveux et offrent ainsi des implications pratiques pour optimiser les après‐shampooings afin d'améliorer l'expérience de l'utilisateur et la santé des cheveux.

## INTRODUCTION

Hair conditioners are formulated with a variety of active ingredients that contribute to conditioning, moisturizing and protecting the hair [1]. The key active components typically include cationic surfactants, humectants, emollients and silicones [2, 3, 4]. Cationic surfactants adhere to the hair surface, providing conditioning benefits by neutralizing hair fibre negative charge. Humectants retain moisture, while emollients lubricate and soften the hair, and silicones form a smooth, protective film that enhances shine and reduces frizz [4]. These ingredients primarily function to reduce friction between hair fibres, thereby minimizing combing force and frizz, and improving manageability. Effective conditioning requires that active ingredients, such as cationic surfactants with positively charged polar groups (e.g., quaternary ammonium), preferentially bind to the negatively charged hair surface rather than interacting with the aqueous phase [1]. This binding process neutralizes the hair's negative charge, leading to conditioning effects like detangling and cuticle scale flattening, which are particularly beneficial for damaged hair [1, 2, 3, 4, 5].

Of the various commonly used cationic surfactants, polyquaterniums are known to form more strongly binding films due to their cationic polymer composition and high molecular weight [1, 6, 7]. These polyquaterniums interact with surfactants in the conditioner formulation, forming polyelectrolyte‐surfactant complexes [4, 5]. However, they may become difficult to wash off, leading to build‐up over time [8]. It is also known that the substantivity of the polyquaternium depends on its molecular weight [7, 9]. Molecules with higher molecular weights tend to exhibit enhanced dispersion bonding and decreased entropy, leading to reduced hydrophilicity and an increased tendency to partition from the aqueous phase to the hair surface F‐layer [3].

It is largely accepted that cationic surfactants, particularly aliphatic quaternary ammonium compounds such as hexadecyltrimethylammonium chloride (CTAC), behentrimonium chloride (BTMC), behentrimonium methosulfate (BTMS) and stearylalkonium chloride (STAC), adhere to the hair surface predominantly through electrostatic interactions [10, 11]. This adhesion is hypothesized to be facilitated by the low isoelectric point (IEP) of hair [10, 12], which makes hair negatively charged at the pH levels typical of conditioner formulations, such as between pH 3.5 and 5.5. Compared to polyquaterniums, cationic surfactants have a lower molecular weight, enabling immediate conditioning and detangling effects without forming films, thereby minimizing the risk of product buildup. In addition to electrostatic interactions, cationic surfactants can also bind to the hair surface through hydrophobic interactions, such as van der Waals forces, simultaneously stabilizing the electrostatic interactions. Longer carbon chains increase the number of hair shaft contact points, resulting in better binding [13]. However, the extent of hydrophobic interactions is generally considered to be less than that observed with polyquaterniums.

Several methods are available to quantify deposition of cationic surfactants on hair, including Inductively Coupled Plasma Optical Emission Spectroscopy (ICP‐OES) [14], Time‐of‐Flight Secondary Ion Mass Spectrometry (ToF‐SIMS) [14], X‐Ray Fluorescence spectroscopy (XRF) [15] and Atomic Force Microscopy [16]. However, these techniques do not provide insight into the deposition kinetics and have limitations in detecting hydrocarbons due to a low signal‐to‐noise ratio, and they are more sensitive to elements with higher atomic numbers such as silicones. While dissipative quartz crystal microbalance (D‐QCM) and ellipsometry can measure deposition kinetics, current technology is limited to using solid flat substrates rather than actual hair [17]. Furthermore, the deposition of substances on hair is notoriously challenging to measure due to its fibrous non‐homogenous cuticle surface. Hair also has a porous keratinous cortex with a propensity to swell. Finally, its natural origins mean enormous variation between every strand, making measurements difficult to reproduce and data challenging to interpret.

Streaming potential is an effective technique to study the sorption process of adsorbates onto macroscopic substrates, including fibrous samples such as hair. It is also able to measure the changes in interactions between electric fields and charged surfaces, allowing quantification of the in situ deposition kinetics of cationic surfactants [18].

In this study, we defined and modelled the deposition process of four different, commonly used cationic surfactants—behentrimonium chloride (BTMC), behentrimonium methosulfate (BTMS), hexadecyltrimethyl ammonium chloride (CTAC), and stearylalkonium chloride (STAC)—on healthy and damaged human hair. Wet combing analysis was conducted to understand the impact on combability. To further validate the model, we compared deposition on a flat silicon oxide wafer to negate the impact of hairs' fibrous nature and sample variations owing to their natural biological origins.

## MATERIALS AND METHODS

### Conditioner formulations

Four conditioner formulations were prepared for the various analyses, consisting of a fatty alcohol mixture, emulsifier, cationic surfactant, preservative and deionized water (Table 1). The oil phase consisted of cetyl/stearyl alcohol (70:30) and was supplied by Sigma‐Aldrich (Saint Louis, Missouri, USA). The emulsifier employed in the formulations was glyceryl stearate (and) PEG‐100 stearate (SP Arlacel 165‐FP MBAL‐PA(RB), Croda, Yorkshire, UK). We obtained different cationic surfactants: behentrimonium chloride (Jeenquat BTMC‐85%) from Vantage Specialty Chemicals (Chicago, Illinois, USA); behentrimonium methosulfate (SP Incroquat Behenyl TMS‐50 MBAL‐PA(MH)) from Croda; and hexadecyltrimethyl‐ammonium chloride and stearylalkonium chloride from Sigma‐Aldrich. The preservative was phenoxyethanol (and) caprylyl glycol (and) sorbic acid (Optiphen Plus, Ashland Inc., Bridgewater, New Jersey, USA). Molecular structures of the surfactants are provided in Figure S1.

**TABLE 1:** Evaluated hair conditioner formulations.

Ingredient	Conditioner 1	Conditioner 2	Conditioner 3	Conditioner 4
Cetyl/stearyl alcohol	4 g	4 g	4 g	4 g
Emulsifier	2 g	2 g	2 g	2 g
Cationic surfactant	1.18 g BTMC (85%)	2 g BTMS (50%)	1 g CTAC (100%)	1 g STAC (100%)
Preservative	1 g	1 g	1 g	1 g
Deionized water	91.82 g	91 g	92 g	92 g
Total	100 g	100 g	100 g	100 g

To prepare the formulations, deionized water with dispersed cationic surfactant was heated to approximately 70°C with stirring in a 250 mL beaker. In a separate 50 mL beaker, the oil phase (cetyl/stearyl alcohol and emulsifier) was melted (while mixing) by heating just above 70°C. The melted oil phase was added to the beaker containing cationic surfactant dissolved in water while mixing and maintaining the temperature at 70°C. The heat source was removed, and during the cooldown phase, preservative was added. The amounts of cationic surfactants added to each formulation varied due to the different stock purities to achieve a 1% cationic surfactant concentration. The pH values are summarized in Table S1.

### Hair tresses for sorption studies

Asian Black blended hair tresses (8 inches in length) were sourced from International Hair Importers & Products Inc. (IHIP, Glendale, New York, USA) and represented in Figure S2. The virgin hair used was standard REMY hair, verified to be free from chemical treatments and denoted as ‘healthy’ in this work. Damaged hair samples were obtained post‐bleach, prepared using IHIP's standardized in‐house bleaching protocol (‘Regular Bleached’). These samples were derived from the same batch as the healthy hair and denoted as ‘damaged’ in this work. As the bleaching process was conducted by IHIP, specific protocol details (e.g., peroxide concentration, exposure time) are unavailable for disclosure. Before use, all hair samples were thoroughly cleaned by immersing them in 500 mL of 1% sodium dodecyl sulfate (SDS, pH 4.9, 25°C) per 1 g hair switch for 30 s with gentle lathering, rinsed under tap water for 30 s, and the procedure repeated 7 times. The hair was then dried at room temperature, weighed and bundled into 1 g tresses without further treatment. The hair tresses were inspected by Scanning Electron Microscope (JEOL JSM‐6701F FEG SEM) to confirm that the cuticles from both healthy and damaged hair remained physically intact, with no exposed cortex and minimal cuticle lifting.

### Silicon wafer

A 150 mm silicon wafer (thickness 0.7 mm) with a silicon oxide coating (1000 Å) was precisely cut into 20 × 10 mm pieces (Siegert Wafer, Aachen, Germany). Before its use for the zeta potential analysis and dynamic streaming potential measurement, the respective pair of Si ∣ SiO_2_ wafer pieces was ultrasonically cleaned with isopropyl alcohol (Ravago Chemicals, Luxembourg) followed by multiple rinses with ultrapure water (Milli‐Q Integral 3, Merck Millipore, Darmstadt, Germany) before drying in an oven at 50°C.

### ζ‐Potential analysis

The ζ‐potential of 1 g hair and a pair of silicon wafer pieces (20 × 10 mm) was measured using a SurPASS 3 instrument (Anton Paar). For hair, the Cylindrical Cell in permeation mode was used, while the Adjustable Gap Cell in tangential mode was employed for silicon wafers. The streaming potential (a DC voltage) was generated as the test solution flowed through a capillary channel (for wafers) or a network of capillaries and pores (for hair fibres). The applied pressure difference (20–60 kPa) created a linear relationship between streaming potential and pressure. The ζ‐potential is calculated from this slope—known as the streaming potential coupling coefficient d*U*_str_/dΔ*p* – using the classic Smoluchowski formula [19]. Normalized ζ‐potential values were calculated by taking ζ divided by the initial ζ‐potential (ζ_o_).

An electrolyte phosphate buffer solution was prepared using 67 mg potassium chloride (KCl), 142 mg sodium hydrogen phosphate (Na_2_HPO_4_) and 28 mg potassium dihydrogen phosphate (KH_2_PO_4_) in 1 L of ultrapure water. These chemicals were obtained from Sigma‐Aldrich (Saint Louis, Missouri, USA).

### Dynamic streaming potential measurement

Adsorption kinetics of the conditioner formulation on hair and silicon wafers was recorded using the SurPASS 3 in dynamic mode. Unlike conventional ζ‐potential analysis, which measures equilibrium conditions, dynamic streaming potential is measured under constant pressure and flow rate, providing real‐time insights into chemical reactions, adsorption and desorption processes at the solid‐water interface. The dynamic streaming potential was recorded with a time resolution of 160 ms and converted to ζ‐potential using the approximated Helmholtz‐Smoluchowski formula (Equation 1).

(1) $$\zeta =\frac{dU_{\mathrm{str}}}{\mathrm{d\Delta}P\;}\frac{\eta }{\varepsilon_{0}\varepsilon_{r}}\kappa_{B},$$

where $U_{\mathrm{str}}$ is the streaming potential, ΔP is the pressure difference across the sample, $\varepsilon_{0}$ is the vacuum permittivity, $\varepsilon_{r}$ and $\kappa_{B}$ are the dielectric constant, the electrolyte viscosity and the conductivity, respectively.

Hair samples or silicon wafers were pre‐soaked in the buffer solution for 15 min to equilibrate before analysis.

#### ζ‐potential as a function of concentration $\zeta =f\left(\text{conc}\right)$

Fresh buffer solutions containing conditioner were sequentially added to the electrolyte (pH≈7.4), with the final conditioner concentration corresponding to 1000 ppm (for hair) and 250 ppm (for silicon wafer). Each addition was allowed to saturate before the next, and streaming potential values were recorded at each saturation point. A stable baseline was established before introducing the conditioner solutions. The pH of the conditioners in the buffer remained stable at pH≈7.4.

#### ζ‐potential as a function of time $\zeta =f\left(t\right)$

The sorption kinetics of the different conditioners on the hair or silicon wafer surfaces were measured by adding 250 ppm conditioner formulation to both systems. A stable baseline was first obtained before the addition, and the streaming potential value was monitored for a total of 2400 s. The data were converted to ζ‐potentials using Equation (1) and then plotted against time.

#### ζ‐potential as a function of pH $\zeta =f\left(\mathrm{pH}\right)$

For hair samples, an approximate pea‐sized (~0.5 cm) amount of each conditioner was gently massaged onto a 1 g wet hair switch for 2 min. Excess conditioner was rinsed off before analysis. The hair switches were then carefully mounted into the sample holder. In the case of silicon wafers, the pH‐dependence experiments were conducted after the final addition of conditioner concentration from the dynamic streaming potential experiment. The pH dependence of ζ‐potential and isoelectric point (IEP) of hair and silicon wafers were determined via automated pH scans. The pH was adjusted in steps of ΔpH = 0.3 using 0.05 mol/L hydrochloric acid (HCl) or potassium hydroxide (KOH), followed by rinsing with 360 mL of solution. ζ‐potential at each pH was measured in quadruplicate, starting from the native pH of the phosphate buffer (pH≈7.4).

### Wet combing experiments

Experiments were carried out on bleached European light brown hair from IHIP with dimensions and mass of: *L* = 18 cm; *W* = 2 cm; *m* = 10 g (including a 2.5 × 2.0 cm wax tab). All hair was shampooed twice with 3% (w/w) sodium laureth sulphate (SLES):cocamidopropyl betaine (CAPB) (12:2) (adjusted to pH 5 with citric acid purchased from Millipore Sigma, Burlington, Massachusetts, USA) before conducting experiments. SLES (Steol CS‐130) and CAPB (Amphosol CA) were obtained from Stepan (Northfield, Illinois, USA). The hair was bleached in two regions of the tress using an acrylic (Acme Plastics, 35 Woodland Park, New Jersey, USA) frame (two pieces) containing two sheets of silicone rubber (McMaster‐Carr, Elmhurst, Illinois, USA) material sandwiched together by the frame. Hair was subjected to a 1‐h bleaching cycle with 120 g of Clairol Professional BW 2 Powder Lightener (The Wella Corporation, Woodland Hills, California, USA) and 147 mL of Salon Care Professional 20 Volume Clear Developer (Arcadia Beauty Labs LLC, Reno, Nevada, USA).

The resulting mixture was applied to damp hair in the window regions of the acrylic frame. The application of bleaching formula to hair was conducted at 25°C and 15 mL of bleaching formula was applied to 0.3 g of hair. Since there were two window treatment regions for each tress, a total of 30 mL of bleaching formula was applied to 0.6 g of hair for each hair tress. At the end of the bleaching cycle, the bleaching formula was rinsed (2 min) from the custom‐designed hair bleaching frame apparatus using an Intellifaucet K250 Mixing Valve (water temperature = 37.8°C; flow rate = 2.5 gallons per minute) purchased from Hass Manufacturing Company (Averill Park, NY, USA) connect to the master water supply. After bleaching, hair was shampooed twice with SLES:CAPB (12:2) (lathered for 30 s) followed by a 30 s rinse cycle. The bleaching treatment uniformity was confirmed visually by the consistency of the hair colour in the bleached regions of the tress in the wet and dry state.

A photograph of an acrylic frame piece alongside a bleached hair tress and a representative figure of a combing curve obtained for a bleached hair tress is provided in Figure S3 and [10]. After bleaching and shampooing, the entire hair tress was treated with 1 g of the conditioning formulation (massaged into the hair for 30 s) and then rinsed for 30 s at the same water temperature and flow rate indicated above.

Wet combing measurements were performed both after bleaching and after treatment with the conditioning formulation using a Dia‐Stron Miniature Tensile Tester (MTT 175) (Dia‐Stron, Ltd., East Anton, Andover, UK). The combing work in the bleached “window” regions of the combing curve was calculated using the Dia‐Stron Windows application software. Measurements were first conducted on untreated, bleached hair, followed by treatment of the entire tress with the conditioning formulation and subsequent combing measurements. The combing curves were analysed by integrating two regions corresponding to the bleached ‘window’ areas and three regions corresponding to undamaged hair (outside the windows). Average values were obtained by measuring two tresses five times each. This approach—using fine hair with bleached window regions—was selected for its ability to detect subtle differences between treatments.

### Attenuated total reflectance fourier transform infrared (ATR‐FTIR) studies

The preparation of hair samples with the respective conditioner formulation followed the same procedure as for the ζ‐potential analysis. Hair was then dried and sent for ATR‐FTIR analysis by TRI Princeton (Spotlight 400, PerkinElmer, with an ATR accessory). Three different tresses per conditioner group were scanned for spectroscopic measurements at three different locations each. The parameters used were an 8 cm^−1^ spectral resolution, 16 scans accumulation within the range of 4000–650 cm^−1^. The absorbance peak at around 1040 cm^−1^ was used to analyse the level of cysteic acid, which was normalized to the absorbance of Amide I (ca. 1650 cm^−1^).

### Data fitting

All graphs were plotted using Python and were fitted according to parameters and equations in Tables S2 and S3. Fitting lines serve as visual guides.

## RESULTS AND DISCUSSION

Both untreated (healthy) and bleached (damaged) hair exhibit a negative ζ‐potential at the intrinsic pH of the buffer solution (pH 7.4, 0.004 M), indicating a negative surface charge. However, the magnitude of this negative charge is significantly different between the two hair types, with healthy hair measuring ζ_healthy_ = −29.2 ± 3.9 mV and damaged hair measuring ζ_damaged_ = −0.9 ± 0.2 mV. While literature on hair ζ‐potential is scarce, the healthy hair value aligns with previous reports [20]. At the pH range of current conditioning systems (3.5 < pH < 5.5), the ζ_healthy_ ranges from ≈−32 mV to near neutral values, and the ζ_damaged_ ranged from ≈−10 to −6 mV, with a minimum at pH≈5. Due to the steep gradient of the ζ‐values in this pH range, the experiments in this work were carried out at pH 7.4.

Bleaching modifies hair structure and surface chemistry, converting cystine disulfide bonds to sulfonic acid groups. This is expected to increase negative surface charge, resulting in a more negative ζ‐potential. However, our results show the opposite trend, with bleached hair exhibiting a less negative ζ‐potential. This unexpected finding suggests complex changes in hair surface properties post‐bleaching.

Several factors contribute to the unexpected decrease in ζ‐potential magnitude for bleached hair. First, bleaching introduces strongly acidic surface groups, enhancing hair hydrophilicity. This correlates with a decrease in ζ‐potential magnitude, as supported by empirical relationships between surface charge density and hydrophilicity [21, 22]. Second, the increased hydrophilicity and structural changes in damaged hair allow greater water penetration, which introduces additional ionic conductance within the hair fibre, previously unaccounted for by the classical Smoluchowski model and Equation (1). Consequently, the true ζ‐potential is underestimated, resulting in an apparent ζ‐potential (ζ_app_). Finally, damaged hair fibres swell, blurring the interface between the solid surface and aqueous solution. This shift from the hair surface to a transition zone weakens the streaming potential signal, distorting the measured ζ‐potential [23]. These combined effects result in the ζ‐potential magnitude of damaged hair being lower than expected at pH 7.4.

A critical distinction arises between the intrinsic surface charge (σ) of hair (a theoretical property determined by its chemical composition) and the ζ‐potential (ζ or ζ_app_), which reflects the effective interfacial potential measurable via electrokinetic techniques. While the intrinsic charge and true surface potential cannot be directly quantified experimentally, ζ and ζ_app_ provide practical insights into the electrostatic environment ‘sensed’ by adsorbates approaching the hair surface and therefore influence the deposition of adsorbates greatly. Although damaged hair has a more negative intrinsic surface charge, this is screened off from the adsorbates who feel a neutral charge (ζ_app_). In contrast, the ζ‐potential of healthy hair more closely resembles the intrinsic surface charge due to its intact surface. This has major implications for deposition affinity to hair and will be discussed in this article.

### Adsorption on healthy and damaged hair $\zeta =f\left(\text{conc}\right)$

**FIGURE 1:**
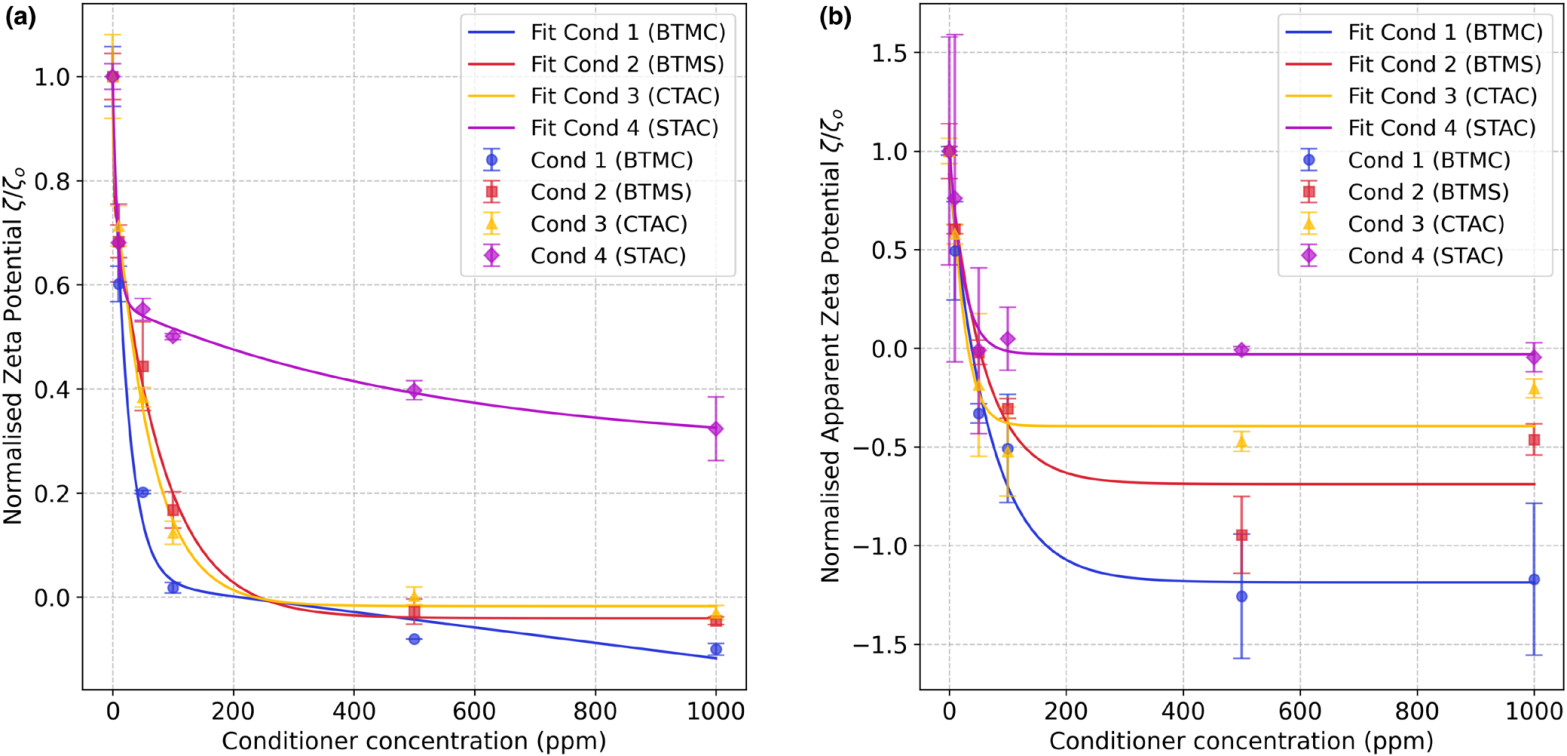
Changes in normalized ζ‐potential with increasing conditioner concentration added to (a) healthy hair, and (b) damaged hair.

Conditioners 1–4, containing 1% of BTMC, BTMS, CTAC and STAC, respectively, were applied to healthy and damaged hair. Data are presented as saturation normalized ζ‐potential to eliminate the differences in starting ζ‐potentials (Figure 1). The absolute ζ‐potentials are presented in Figure S4.

Conditioner 4 (STAC) induced the least change in ζ‐potential, while Conditioner 1 (BTMC) caused the most significant change for both healthy and damaged hair. The limited deposition of Conditioner 4 (STAC) can be attributed to the presence of the aromatic ring at the terminal end of STAC structure, causing steric hindrance and preventing electrostatic attraction between the cationic group and the negatively charged hair surface.

Conditioners 1 (BTMC) and 4 (STAC) did not reach saturation on healthy hair, even at concentrations as high as 1000 ppm (equivalent to 10 ppm of cationic surfactant), while the other conditioners achieved equilibrium at around 500 ppm. On damaged hair, however, all four conditioners saturated by 500 ppm, with Conditioners 3 (CTAC) and 4 (STAC) saturating at the lowest concentrations (<200 ppm). This lower saturation concentration for damaged hair arises from two interrelated factors: early charge reversal and limited hydrophobic stabilization. Despite damaged hair's higher intrinsic negative surface charge due to cysteic acid groups, its apparent ζ‐potential (ζ_app_) is less negative compared to healthy hair. The weaker ζ_app_ allows charge reversal to be achieved at lower conditioner concentrations. Once the ζ‐potential reverses, electrostatic repulsion prevents further ionic adsorption. Beyond charge reversal, deposition relies mostly on hydrophobic interactions. For Conditioners 3 (CTAC, short C16 chain) and 4 (STAC, bulky aromatic group), these interactions are inefficient due to reduced hydrophobicity and steric hindrance, preventing stable multilayer formation.

In contrast, healthy hair has a more negative ζ‐potential value, delaying charge reversal and enabling prolonged electrostatic adsorption till higher conditioner concentrations. This difference in charge reversal timing explains the saturation gap whereby damaged hair becomes self‐limiting (due to repulsion) at lower concentrations, while healthy hair sustains adsorption until higher thresholds. Studies have reported that damaged hair enhances attraction to cationic adsorbates [24], whereas other studies report conflicting findings [2, 16], which could be due to different measurement parameters. However, our data highlight how electrostatic factors can influence saturation behaviour and suggest that damaged hair ultimately binds less total conditioner than healthy hair at neutral pH, as evidenced by its lower saturation concentration.

### Adsorption and pH dependence $\zeta =f\left(\mathrm{pH}\right)$

**FIGURE 2:**
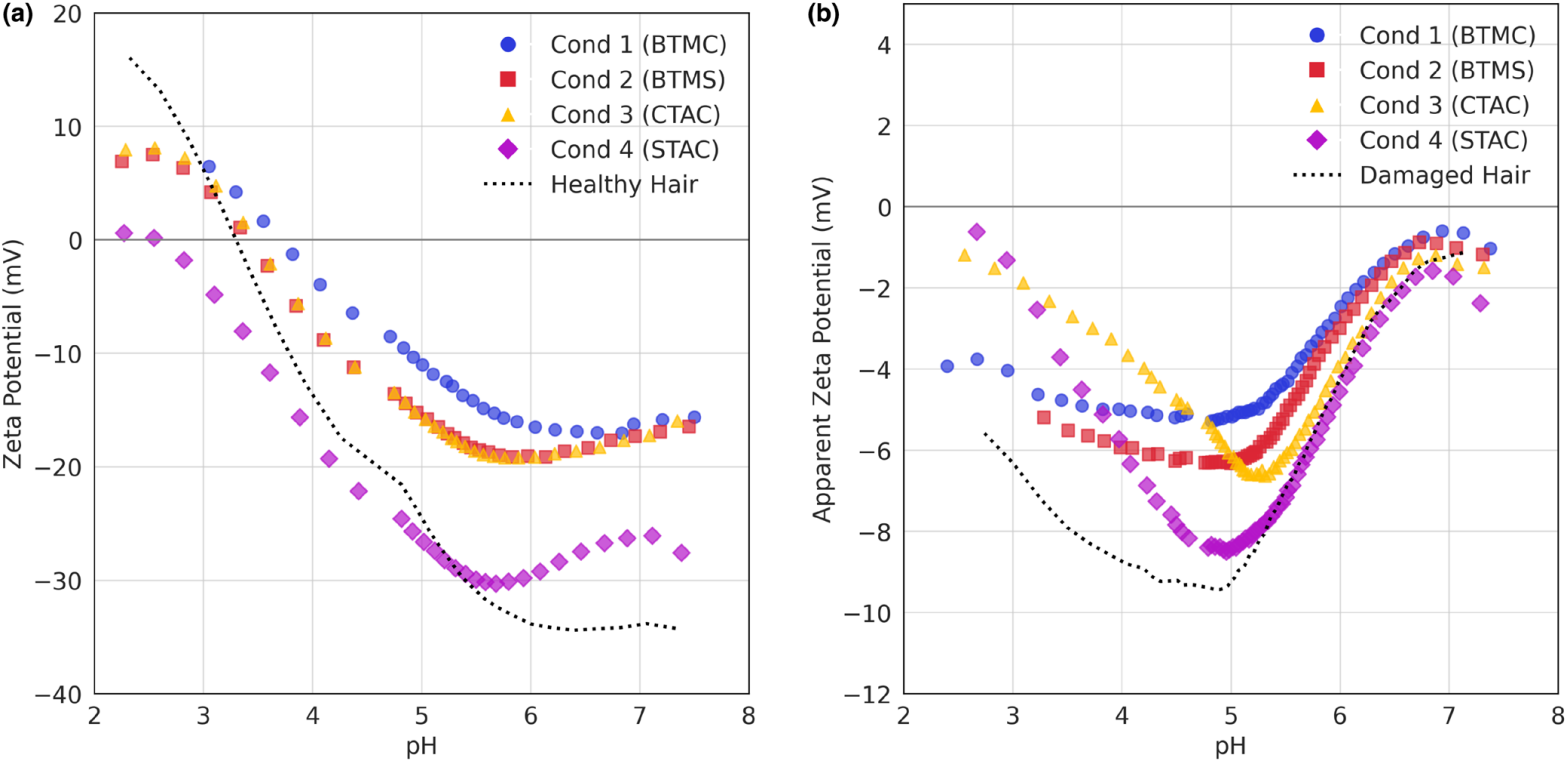
ζ‐potentials with changing pH for (a) healthy and (b) damaged hair applied with different conditioners by hand.

The ζ‐potentials of healthy and damaged hair at different pH were measured before and after conditioner application (Figure 2). In both healthy and damaged hair, the ζ‐potential curves shifted upwards at all pH values and maintained a similar curve shape post‐conditioner addition. The magnitude of the upward shift correlates with the amount of conditioner adsorbed onto the hair surface, which increases in the order of Conditioner 4 (STAC) < Conditioner 3 (CTAC)≈Conditioner 2 (BTMS) < Conditioner 1 (BTMC), similar to the observations in Figure 1.

The IEPs for healthy hair increased in the series of Conditioner 4 (2.58) < Conditioner 2 (3.42)≈Conditioner 3 (3.47) < Conditioner 1 (3.70). Considering an uncertainty of ±0.2 pH units for the IEP, the ζ‐potential analysis cannot distinguish the effects of Conditioners 2 (BTMS) and 3 (CTAC) at equilibrium. However, the IEP shift to higher pH values implies the cationic surfactants coated the hair surface and reduced its negative surface charge, requiring a lower concentration of hydrogen ions (H^+^) to neutralize. The lower IEP of Conditioner 4 (2.58) compared to healthy hair (3.31) suggests that, despite the low levels of cationic STAC remaining on the surface after rinsing, other formulation ingredients have adsorbed onto the hair surface, causing the IEP to shift more acidic. The adsorbates' effect remains at higher pH values (pH > 5.5), indicating Conditioner 4 (STAC) was not completely removed by rinsing. The slight swelling observed around pH 5.5 with all conditioners was likely induced by other formulation ingredients, a phenomenon not observed in the original healthy hair sample.

For damaged hair, the change in ζ‐potential values at neutral pH was less pronounced, with a slight upward shift for Conditioners 1 (BTMC), 2 (BTMS) and 3 (CTAC) and a minimal shift for Conditioner 4 (STAC). Notably, the bare damaged hair curve exhibited a minimum around pH 5, indicative of fibre swelling. However, the application of conditioners, particularly Conditioners 1 and 2, reduced the prominence of this minimum, suggesting that the conditioners coat the hair fibre surface and hinder water penetration. Despite treatment with any conditioner, damaged hair failed to reach IEP.

### Adsorption and carbon chain length

The enhanced deposition of Conditioner 1 (BTMC) and 2 (BTMS) can be attributed to the longer carbon chain length of the behenyl group (C22), compared to the cetyl (C16) and stearyl (C18) groups in Conditioner 3 (CTAC) and Conditioner 4 (STAC), respectively. The longer carbon chain increases substantivity, promoting stronger interactions with the hair surface. When comparing Conditioner 1 (BTMC) and Conditioner 2 (BTMS), the smaller chloride ions in Conditioner 1 dissociate more readily from the behenyl group than the larger methosulfate anions in Conditioner 2. This results in Conditioner 1 having a higher effective charge density, enhancing its hair surface interaction. Additionally, the methosulfate anion is more hydrophilic than the chloride anion, increasing the BTMS aqueous solubility and reducing its hair surface affinity. This self‐emulsifying property improves formulation stability [25] but may limit its hair deposition.

As both BTMC and CTAC in Conditioners 1 and 3 possess chloride anions, the influence of carbon chain length on deposition is isolated. The longer behenyl (C22) chain in BTMC enables stronger van der Waals interactions with the hair surface, promoting self‐association and enhancing deposition. Additionally, the longer chain length reduces Conditioner 1 solubility, increasing its partitioning toward the hydrophobic hair surface. This longer chain length also contributes to higher formulation viscosity, potentially prolonging the residence time of the conditioner on the hair fibres compared to shorter‐chain cations [26]. However, the comparison between Conditioner 3 (C16) and Conditioner 4 (C18) reveals an opposite trend, with CTAC depositing more than STAC. This is due to the presence of the bulky STAC aromatic group introducing steric hindrance and blocking hair shaft interactions despite its longer carbon chain length.

### Adsorption and desorption dynamics $\zeta =f\left(t\right)$

**FIGURE 3:**
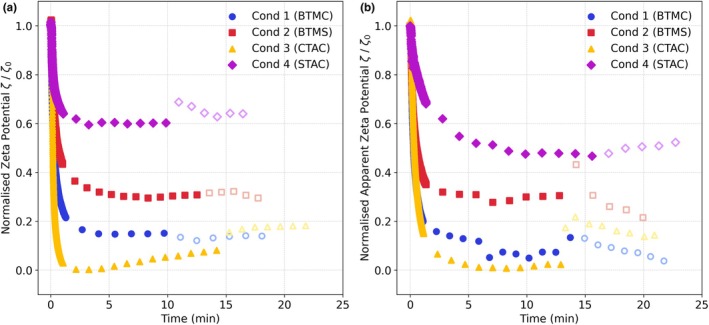
Normalized sorption kinetics of (a) healthy hair, and (b) damaged hair when the four conditioners (250 ppm) were added into the buffer solution and upon desorption with buffer solution. Filled markers represent adsorption data, empty markers represent desorption data. Conditioners were added at *t* = 0.

Hair switch ζ‐potential was measured over time (Figure 3). In general, the deposition of all four conditioners was quick on both healthy and damaged hair, obtaining equilibrium within the first 5 min.

Conditioner 4 (STAC) induced a significantly smaller zeta shift on both healthy and damaged hair (Figure 3). The overall ζ‐potential shift upon conditioner addition was less pronounced for damaged compared to healthy hair, with no apparent charge reversal either case (Figure S4). Interestingly, the sorption experiments revealed that Conditioner 3 (CTAC) adsorbed more strongly on both healthy and damaged hair than Conditioners 1 and 2, contrary to the trends in Figure 1. This could be due to the shorter C16 chain length of CTAC and the higher mobility in solution compared to the longer carbon chains, allowing faster diffusion to the hair surface and more rapid adsorption [27]. However, this strong initial adsorption was followed by a gradual decline, particularly evident in healthy hair (Figure 3a), most likely due to the weaker van der Waals interactions stabilizing CTAC molecules on the hair surface. This decline may also be due to surfactant micelle formation which solubilizes and removes previously deposited surfactants, though to a lesser extent. Although the conditioner concentration used (250 ppm, equivalent to ≈0.00025% w/w CTAC) was well below the CTAC critical micelle concentration (CMC) (0.03%–0.038% w/w [27, 28]), the presence of fatty alcohols and other formulation ingredients likely reduced the effective CMC by acting as co‐surfactants and stabilizers, facilitating mixed micelle formation [29, 30]. Micelles also form at lower concentrations for shorter chain length surfactants.

During rinsing, Conditioners 3 (CTAC) and 4 (STAC) were more easily removed from healthy hair, with Conditioner 3 also easily removed from damaged hair. This can be attributed to the shorter carbon chains of CTAC and STAC, resulting in weaker van der Waals forces. The shear forces exerted by the rinse liquid were sufficient to disrupt any bilayer or multilayer structures on the hair surface [24]. Nevertheless, after multiple rinse cycles, the ζ‐potential stabilized at a consistent value across all conditions, indicating that the electrostatic attraction between the cationic surfactant heads and the negatively charged hair surface was strong enough to withstand the rinse shear forces. In contrast, on healthy hair, Conditioners 1 (BTMC) and 2 (BTMS) showed negligible change in ζ‐potential upon rinsing, suggesting minimal conditioner removal, highlighting the varying adsorption and retention behaviours influenced by their molecular structures and hair surface interactions.

### Comparison to a model surface

Hair's complex fibrous structure complicates adsorption analysis by surface‐sensitive techniques such as streaming potential. To simplify this complication, silicon oxide wafers were used to mimic the negative surface charge of human hair as a model system. The rationale for employing silicon oxide wafers lies in their ability to provide a controlled, homogeneous surface that isolates electrostatic interactions—free from the confounding variables inherent to hair's fibrous morphology (e.g., cuticle layering, chemical heterogeneity and variable surface area). The wafer's smooth, reproducible surface allows systematic evaluation of surfactant adsorption trends under standardized conditions, which is critical for mechanistic interpretation. A summary of their similarities and differences is documented in Table S4.

Under the same experimental conditions as the healthy and damaged hair studies, the silicon oxide wafer exhibited a ζ‐potential of ζ = −54.6 ± 8.6 mV, significantly more negative than healthy hair. The smaller surface area of silicon oxide wafers compared to hair fibres necessitated lower conditioner concentrations (250 ppm) for streaming potential measurements than for hair fibres.

### ζ and concentration

**FIGURE 4:**
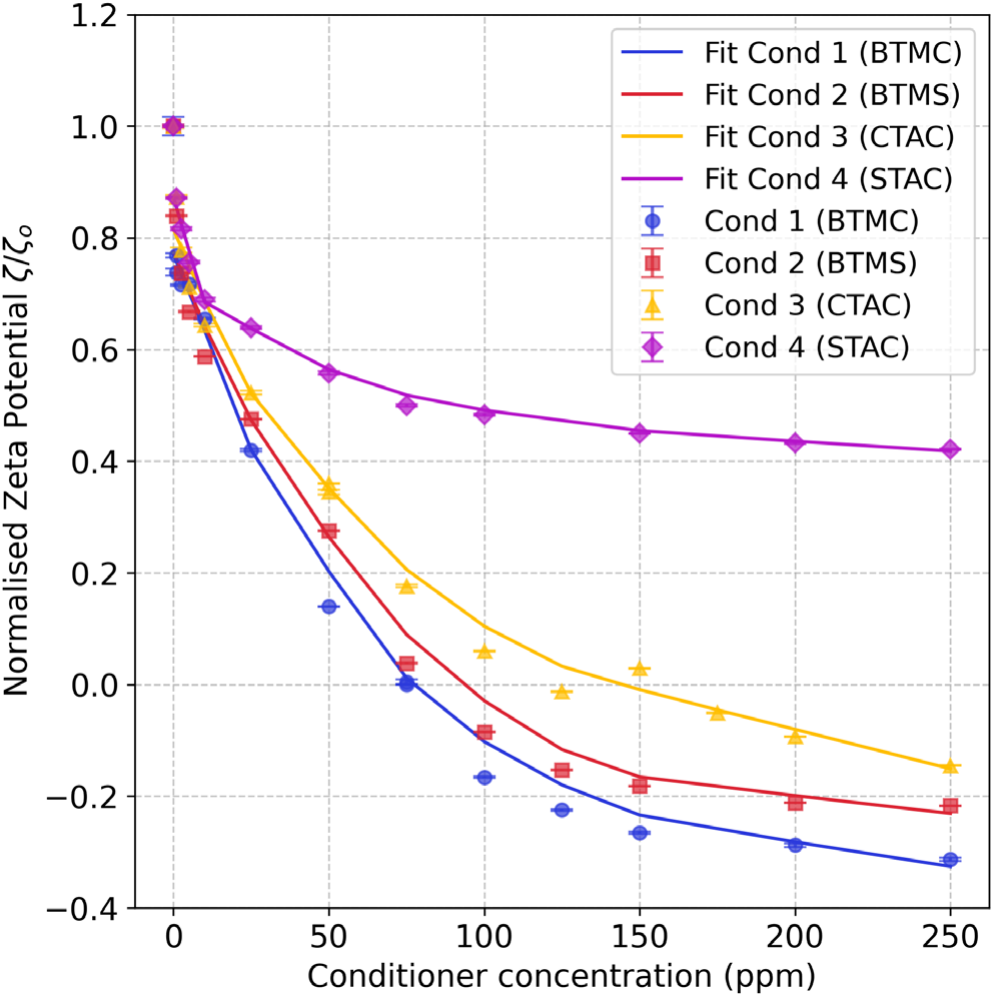
Changes in normalized ζ‐potential with increasing conditioner concentration added to Si | SiO_2_ wafer.

Despite differences in surface chemistry, ζ‐potential and surface areas, the trend in affinity of Conditioners 1–4 observed for the silicon oxide surface (Figure 4) was consistent with those observed for both healthy and damaged hair (Figure 1). The lower surfactant concentration required for charge reversal on silicon wafers (75–150 ppm) relative to hair (≈200 ppm) can be attributed to the wafers' smaller surface area (4 cm^2^ vs. ≈342 cm^2^ for hair) and their smooth, homogeneous surface, which facilitates more efficient surfactant adsorption (Figures S4a and S6). Although hair has a less negative ζ‐potential, its complex, fibrous structure and chemical heterogeneity reduce surfactant adsorption efficiency, requiring a slightly higher concentration to achieve charge reversal. The higher positive ζ‐potential observed for wafers at equilibrium (≈10 mV vs. 0–2 mV) further confirms the more complete surface coverage. This difference may arise from the chemical homogeneity of the silicon oxide coating compared to the complex, tangled structure of individual hair strands in a hair plug.

### ζ and pH

**FIGURE 5:**
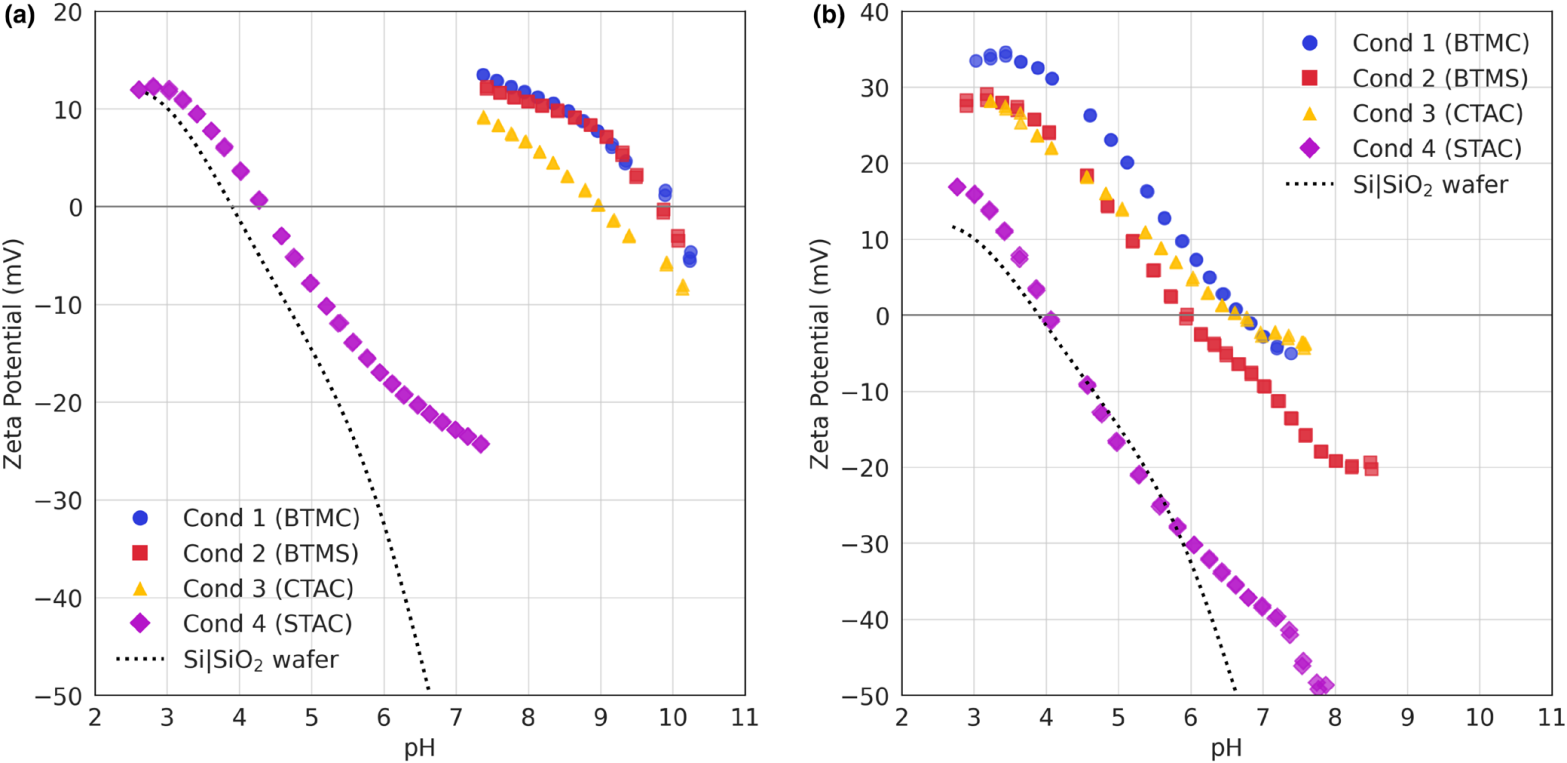
ζ‐potential changes of Si | SiO_2_ wafer with pH levels at (a) adsorption equilibrium (250 ppm) and (b) after desorption.

The silicon oxide wafer pieces were characterized by performing a pH scan of the ζ‐potential at 250 ppm of each conditioner formulation (Figure 5). This pH dependence has been extensively studied in various buffer solutions [31]. The IEPs increased in the following order: pristine wafer (3.91) < Conditioner 4 (4.32) < Conditioner 3 (8.99) < Conditioner 2 (9.84)≈Conditioner 1 (9.98). Given the uncertainty of ±0.2 pH units for IEP, ζ‐potential analysis could not distinguish between Conditioner 1 (BTMC) and Conditioner 2 (BTMS) at adsorption equilibrium (Figure 5a). The adsorption effects of Conditioner 4 (STAC) on the silicon oxide surface were also not significantly different from those of the pristine wafer. However, the increasing divergence in ζ‐potential between Conditioner 4 and the pristine wafer at pH values above the IEP clearly indicates the partial adsorption of the cationic surfactant STAC.

After rinsing once with buffer solution, some desorption of reversibly bound cationic surfactant occurred (Figure 5b) and the ζ‐potential of all modified silicon wafer samples becomes negative at neutral pH. The IEPs increased in the following order: pristine wafer (3.91)≈Conditioner 4 (4.03) < Conditioner 2 (5.95) < Conditioner 3 (6.68)≈Conditioner 1 (6.72). Titration curves for silicon wafers treated with Conditioners 1–3 merge, with their IEPs shifting toward neutral pH. The most significant change in IEP occurred for Conditioner 2 (3.89) followed by Conditioner 1 (3.26). The IEP for Conditioner 2 (5.95) becomes lower than that of Conditioner 3 (6.68), which now closely resembles Conditioner 1 (6.72). The IEP for the silicon oxide surface treated with Conditioner 4 (4.03) approaches that of the pristine wafer (3.91), as does the ζ‐potential across the remaining pH range. This suggests that Conditioner 4 (STAC) is almost completely removed during rinsing. The pH dependence of the ζ‐potential and the IEPs is consistent with the results observed after applying the conditioners to healthy hair (Figure 2a). The IEPs are summarized in Table S5.

### ζ and time

**FIGURE 6:**
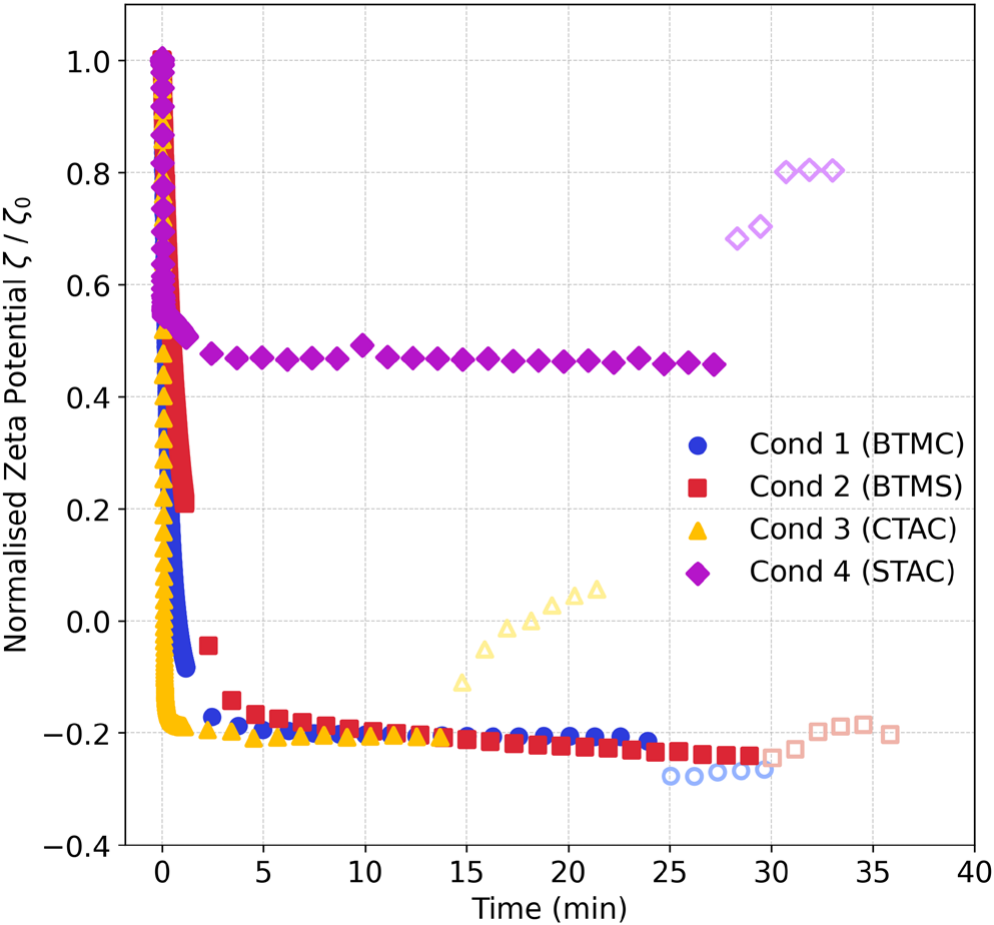
Normalized sorption kinetics of Si | SiO_2_ wafer when Conditioners 1–4 (250 ppm) were added into the buffer solution and upon desorption with buffer solution. Filled markers represent adsorption data, while empty markers represent desorption data. Conditioners were added at *t* = 0 min. Data was normalized to ζ at *t* = 0.

Sorption kinetics were also evaluated on silicon wafers (Figure 6 and Figure S7). The adsorption and rinse behaviours were consistent with those for healthy and damaged hair (Figure 3 and Figure S5). Briefly, Conditioners 3 (CTAC) and 4 (STAC) exhibited the highest tendency to be removed by rinsing relative to Conditioners 1 (BTMC) and 2 (BTMS). Although less pronounced than for healthy and damaged hair, Conditioner 3 (CTAC) exhibited stronger initial adsorption on wafers relative to the other three, despite smaller ζ‐potential changes than Conditioners 1 (BTMC) and 2 (BTMS, Figure 4). This behaviour aligns with the trends observed for both healthy and damaged hair (Figure 3), though to a lesser extent.

While the wafer serves as a qualitative model for electrostatic interactions, it fails to quantitatively replicate hair's charging behaviour due to factors like silanol group reactivity, smaller surface area and susceptibility to contamination (e.g., airborne hydrocarbons), which were mitigated through a rigorous cleaning procedure, as detailed in Materials and Methods. Nevertheless, the wafer model's ability to reproduce the relative adsorption trends of cationic surfactants underscores its utility as a screening tool for formulation development, particularly when prioritizing electrostatic‐driven interactions over hair‐specific structural effects.

The similarities and differences of the silicon wafer model are summarized in Table S4, emphasizing its value for comparative studies while acknowledging its inability to fully replicate hair's charging behaviour.

### Wet combing

**FIGURE 7:**
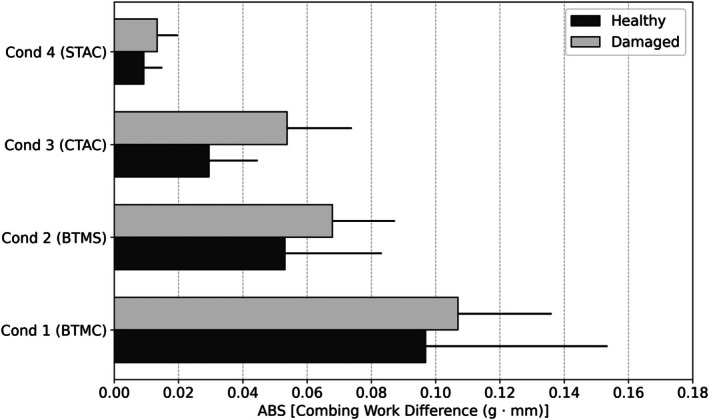
Combing work data (integrated window regions) obtained from wet combing curves for hair treated with various conditioner formulations.

To better understand the effects of different cationic surfactants and correlate their deposition properties with conditioning efficacy, we investigated the wet combing properties of hair treated with the various conditioners (Figure 7). Wet combing forces, influenced by fibre swelling and adhesion, are significantly reduced by most conditioning agents, partly due to the surface tension properties of the treatments [32].

As in accepted practice, fine hair (dark brown) was used to minimize error associated with wet combing measurements, and damaged hair was used to increase the measurement dynamic range. It should be noted that the streaming potential data presented thus far were measured using bleached and virgin Asian black hair. However, the effects of bleaching and subsequent treatment with cationic guar on the streaming potential profiles (data not shown) only found differences in the magnitude of the various parameters as compared to virgin hair [10].

Combing work difference is defined as the difference between the combing work after and before conditioner treatment. Overall, all treatments effectively conditioned both healthy and damaged (bleached) hair. For both healthy and damaged conditions, the reduction in combing force difference was Conditioner 4 (STAC) < Conditioner 3 (CTAC) < Conditioner 2 (BTMS) < Conditioner 1 (BTMC). Damaged hair has slightly higher combing work differences than healthy hair across all conditioners, suggesting that conditioners have a more pronounced effect on damaged hair, though these differences were not significant.

BTMC is commonly accepted to provide better combing force reduction than BTMS; though the mechanism behind this difference in performance is not fully understood. As the key difference between BTMS and BTMC lies only in their counterion, we propose that Conditioner 1 (BTMC)'s better conditioning effect is likely due to the smaller chloride ion being more easily displaced, enhancing electrostatic interactions with the negatively charged hair surface. In contrast, the larger methosulfate ion in Conditioner 2 (BTMS) hinders this process. This explanation aligns with the results obtained from our streaming potential analyses.

It is generally accepted that longer chain cationic surfactants provide greater conditioning benefits, including reduction in wet combing forces, compared to shorter chain analogues [2, 10, 33]. This phenomenon is attributed to the increased stability provided by van der Waals interactions in longer‐chain surfactants. While electrostatic interactions are the primary driving force for cationic surfactant‐hair surface interactions, these interactions are stabilized by van der Waals forces between adjacent long alkyl chains. For instance, shorter‐chain cationic surfactants (with fewer than 10 carbon atoms) do not provide appreciable reductions in combing force. Similarly, Conditioner 3 (CTAC) and Conditioner 4 (STAC) produce less wet combing force reduction than Conditioner 1 (BTMC) and 2 (BTMS). Contrary to expectations, Conditioner 3 provided greater combing force reduction than Conditioner 4, likely due to the higher deposited amounts of CTAC compared to STAC, as evidenced by streaming potential analyses.

### Damage assessment by ATR‐IR

ATR‐IR is often used to measure the hair surface cysteic acid level as an indication of oxidative damage. Bare healthy hair has much lower oxidative damage (2.1 ± 0.1) compared to bare damaged hair (7.2 ± 0.1) (Figure S8). Despite improvement in combability and the variation in conditioner deposition, there was no appreciable change in cysteic acid levels of healthy and damaged hairs after the application of any conditioner, clearly indicating that applying conditioner does not reverse any oxidative damage on hair.

### Practical implications for hair care formulations

While this study was conducted at neutral pH to ensure reliable streaming potential measurements, the findings offer critical insights for real‐world hair care products, which are typically formulated at acidic pH levels (3.5–5.5). These pH‐dependent effects are particularly relevant given the distinct behaviours of healthy and damaged hair. For healthy hair, at pH 3.5–5.5, the ζ‐potential becomes less negative as it approaches its IEP, reducing electrostatic attraction to cationic surfactants. This weakens deposition, especially for short‐chain surfactants like CTAC (C16), which rely heavily on ionic interactions. However, long‐chain surfactants like BTMC (C22) can partially compensate through hydrophobic tail interactions, retaining moderate adsorption even in acidic conditions. Damaged hair retains a negative ζ‐potential even at pH 3.5–5.5. This sustains the electrostatic binding with cationic surfactants, enabling efficient deposition comparable to neutral pH. Additionally, mild swelling of damaged hair at pH~5 may increase accessibility to binding sites, potentially enhancing surfactant penetration.

These differences highlight the need for tailored formulations whereby acidic formulations (pH 3.5–5.5) can leverage both electrostatic and hydrophobic interactions for effective deposition of long‐chain surfactants, and shorter‐chain surfactants or pH‐balanced systems may be preferred for healthy hair to prevent over‐deposition.

## CONCLUSIONS

This study demonstrates the use of streaming potential as a powerful tool for characterizing the adsorption behaviour of cationic surfactants on human hair. The findings show that ζ‐values, including ζ_app_, and to a lesser extent the intrinsic charge, govern adsorption behaviour, especially for damaged hair. Damaged hair's structural defects create a distinction between its highly negative intrinsic charge and the interfacial potential sensed by adsorbates, with direct implications for conditioner formulation strategies targeting hair of different damage levels. A direct comparison between healthy and damaged hair was limited by challenges in controlling variables such as differing zeta potentials (apparent vs. true) and structural factors like swelling.

By analysing changes in ζ‐potential, we quantified differences in adsorption and substantivity among four cationic surfactants—BTMC, BTMS, CTAC and STAC—on both healthy and damaged hair. These findings were used to interpret ‘real‐world’ combing force studies and model the forces driving surfactant‐hair interactions.

The results revealed that BTMC exhibited the strongest adsorption, as evidenced by the largest shift in ζ‐potential, followed by BTMS, CTAC and STAC. This was attributed to the combined effects of carbon chain length and counterion size. A longer carbon chain in BTMC enhanced van der Waals interactions with the hair surface, while the smaller chloride counterion facilitated stronger electrostatic interactions. In contrast, STAC showed the weakest adsorption due to steric hindrance from its bulky aromatic group, reducing its affinity for the hair surface. The study also demonstrated how streaming potential measurements could assess sorption kinetics, showing that shorter carbon chain lengths increase diffusivity but decrease substantivity to the hair surface compared to longer chain lengths.

Silicon oxide wafers were shown to be an effective model system to validate streaming potential measurements and provide qualitative insights into surfactant adsorption, confirming the trends observed in hair. Their use was motivated by the need to decouple electrostatic adsorption mechanisms from hair's structural complexity, enabling controlled comparisons of surfactant behaviour. While the model cannot fully replicate hair's charging dynamics (e.g., lower ζ‐potential, surface area effects), it offers a practical platform for rapid screening of cationic surfactants.

Wet combing analysis confirmed that longer chain cationic surfactants, particularly BTMC, significantly reduced wet combing force, highlighting the importance of van der Waals interactions in stabilizing the conditioning film and improving hair manageability.

While the conditioners improved hair combability, ATR‐IR analysis indicated that they did not reverse oxidative damage, as measured by cysteic acid levels, clearly indicating that while conditioners provide immediate benefits to hair's appearance and manageability, they do not repair underlying damage. While this study focused on neutral pH conditions, the mechanistic insights into surfactant adsorption—such as the roles of chain length, counterion size and electrostatic/hydrophobic interactions—provide a predictive framework for behaviour across pH ranges. These findings highlight the necessity of customizing formulations to hair condition: damaged hair benefits from cationic surfactants with long alkyl chains (e.g., BTMC) in acidic environments, whereas healthy hair may require milder, pH‐adjusted systems. By integrating streaming potential analysis into formulation workflows, developers can rationally design conditioners that balance deposition, substantivity and sensory appeal for diverse hair types.

In conclusion, this study highlights streaming potential as a valuable technique for measuring cationic surfactant adsorption on hair, offering insights into molecular interactions governing adsorption, substantivity and rinsability. Though beyond the scope of this work, further studies could include dry combing data in the context of leave‐in formulations or products designed for dry hair management. Nevertheless, these findings advance our understanding of cationic surfactant behaviour and provide practical guidance for optimizing hair care formulations. Furthermore, by leveraging streaming potential, formulators can better predict and enhance the performance of conditioning agents, ultimately improving the efficacy of hair care products.

## CONFLICT OF INTEREST STATEMENT

The authors declare that they have no known competing financial interests or personal relationships that could have influenced the work reported in this paper.

## Supporting information


Appendix S1.

## Data Availability

The data that support the findings of this study are available from the corresponding author upon reasonable request.
