# Supplementary material for: Electrokinetic analysis reveals common conditioner ingredient interactions with human hair
Source: Int J Cosmet Sci. 2025 Oct 22;48(2):379–93. doi: 10.1111/ics.70038 (PMC13068045; doi:10.1111/ics.70038)
Supplement: Supplementary file 1 — Appendix S1. [file ICS-48-379-s001.docx]

**Supplementary Information**


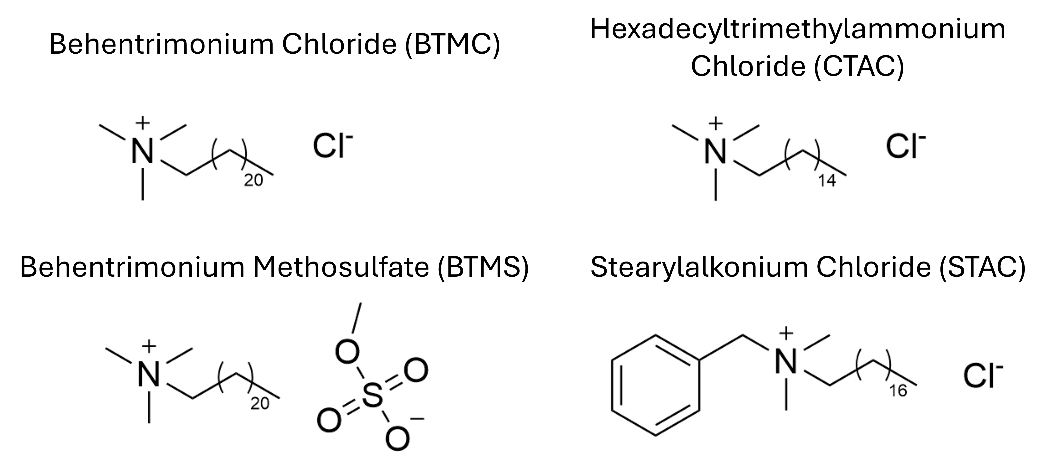


Figure S1. Molecular structures of behentrimonium chloride (BTMC), behentrimonium methosulfate (BTMS), hexadecyltrimethylammonium chloride (CTAC) and stearylalkonium chloride (STAC).


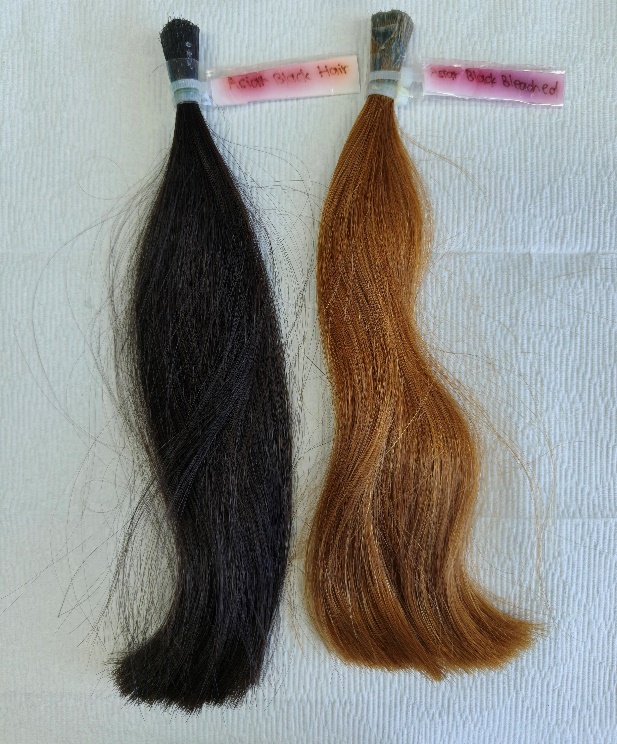


Figure S2: (Left) Asian black (healthy) hair, (Right) Regular Bleached Asian black (damaged) hair.

| **Solution** | **pH (in buffer)** | **pH (in water)** |
| --- | --- | --- |
| Ultrapure water | - | 6.40 |
| 1% SDS in water | - | 4.89 |
| Electrolyte buffer | 7.42 | - |
| Conditioner #1 (up to 2,500 ppm) in buffer | 7.39 | 5.00 |
| Conditioner #2 (up to 2,500 ppm) in buffer | 7.41 | 4.93 |
| Conditioner #3 (up to 2,500 ppm) in buffer | 7.39 | 4.75 |
| Conditioner #4 (up to 2,500 ppm) in buffer | 7.39 | 5.11 |

Table S1: Summary of pH values of the various solutions used.


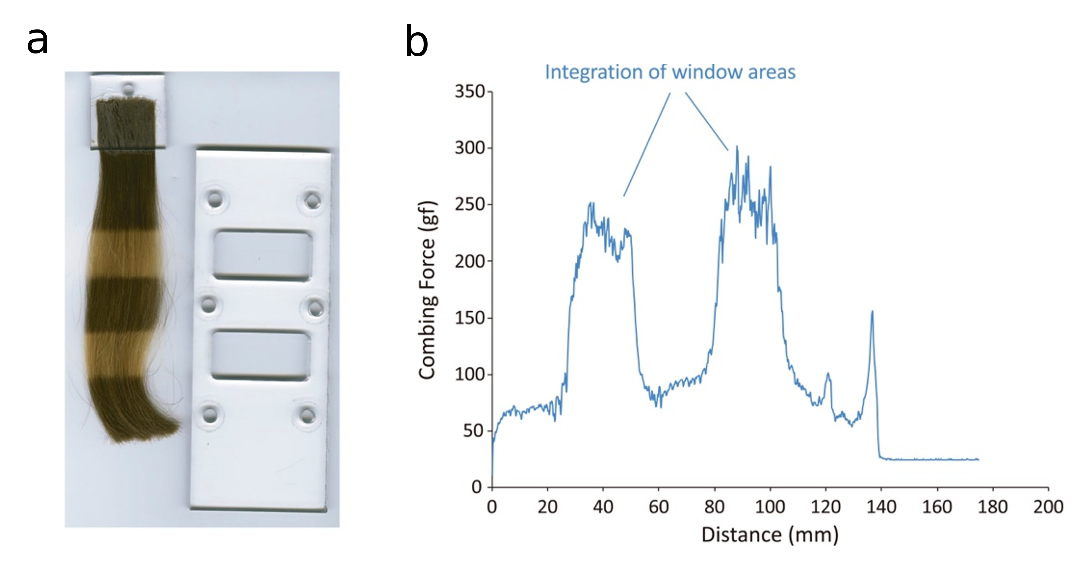


Figure S3: a) Photograph of a hair tress after bleaching with a window treatment frame. Note that only one part of the window treatment frame (one of the acrylic portions) is shown in the photograph. b) Wet combing curve of hair after the bleaching treatment.

Normalised data

| **Hair Type** | **Conditioner** | **Fitting Function** | **Equation** | **Parameters** |
| --- | --- | --- | --- | --- |
| Healthy | #1 | Linear + Exponential Decay | $f\left( x \right)=a\cdot e^{-bx}+c\cdot x+d$ | [0.94, 0.04, -0.00, 0.03] |
|  | #2 | Double Exponential Decay | $f\left( x \right)=a\cdot e^{-bx}+c\cdot e^{-dx}+e$ | [0.21, 2.27, 0.83, 0.01, -0.04] |
|  | #3 | Double Exponential Decay | $f\left( x \right)=a\cdot e^{-bx}+c\cdot e^{-dx}+e$ | [0.15, 2.40, 0.87, 0.02, -0.02] |
|  | #4 | Sigmoid | $f\left( x \right)=\frac{a}{1+e^{-b(x-c)}+d}$ | [0.43, 0.13, 0.28, 0.00, 0.28] |
| Damaged | #1 | Sigmoid | $f\left( x \right)=\frac{a}{1+e^{-b(x-c)}+d}$ | [-6030.75, 0.01, -551.59, 6029.56] |
|  | #2 | Sigmoid | $f\left( x \right)=\frac{a}{1+e^{-b(x-c)}+d}$ | [-4510.04, 0.02, -475.27, 4509.36] |
|  | #3 | Sigmoid | $f\left( x \right)=\frac{a}{1+e^{-b(x-c)}+d}$ | [-3.80, 0.05, -11.03, 3.40] |
|  | #4 | Double Exponential Decay | $f\left( x \right)=a\cdot e^{-bx}+c\cdot e^{-dx}+e$ | [0.80, 0.04, 0.27, 0.04, -0.03] |
| Wafer | All | LOWESS Smoothing | Non-parametric smoothing | N.A. |

*Table S2: Fitting equations and fitting parameters used for plotting normalised zeta potential best-fit lines.*

Zeta and apparent zeta potential values

| **Hair Type** | **Conditioner** | **Fitting Function** | **Equation** | **Parameters** |
| --- | --- | --- | --- | --- |
| Healthy | #1 | Linear + Exponential Decay | $f\left( x \right)=a\cdot e^{-bx}+c\cdot x+d$ | [-28.35, 0.04, 0.00, -0.93] |
|  | #2 | Exponential Decay | $f\left( x \right)=a\cdot e^{-bx}+c$ | [-26.14, 0.02, 0.91] |
|  | #3 | Exponential Decay | $f\left( x \right)=a\cdot e^{-bx}+c$ | [-24.58, 0.02, 0.27] |
|  | #4 | Linear + Exponential Decay | $f\left( x \right)=a\cdot e^{-bx}+c\cdot x+d$ | [-15.96, 0.11, 0.01, -18.43] |
| Damaged | #1 | Sigmoid | $f\left( x \right)=\frac{a}{1+e^{-b(x-c)}+d}$ | [4387.79, 0.01, -562.53, -4387.06] |
|  | #2 | Sigmoid | $f\left( x \right)=\frac{a}{1+e^{-b(x-c)}+d}$ | [3.69, 0.03, -10.72, -3.30] |
|  | #3 | Sigmoid | $f\left( x \right)=\frac{a}{1+e^{-b(x-c)}+d}$ | [3.06, 0.05, -11.03, -2.74] |
|  | #4 | Double Exponential Decay | $f\left( x \right)=a\cdot e^{-bx}+c\cdot e^{-dx}+e$ | [0.33, 7.63, -1.18, 0.03, -0.02] |
| Wafer | All | LOWESS Smoothing | Non-parametric smoothing | N.A. |

*Table S3: Fitting equations and fitting parameters used for plotting absolute zeta potential best-fit lines.*


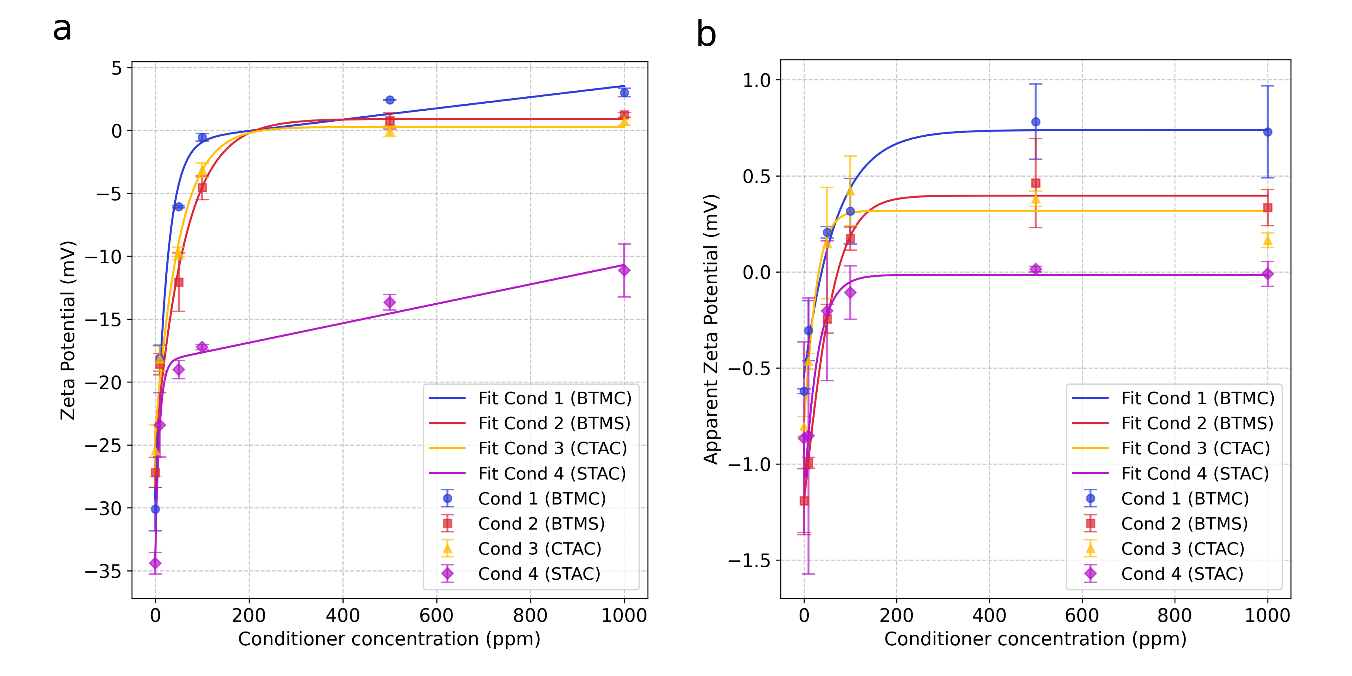


Figure S4: Changes in zeta potential with increasing conditioner concentration added to a) healthy hair, and b) damaged hair.


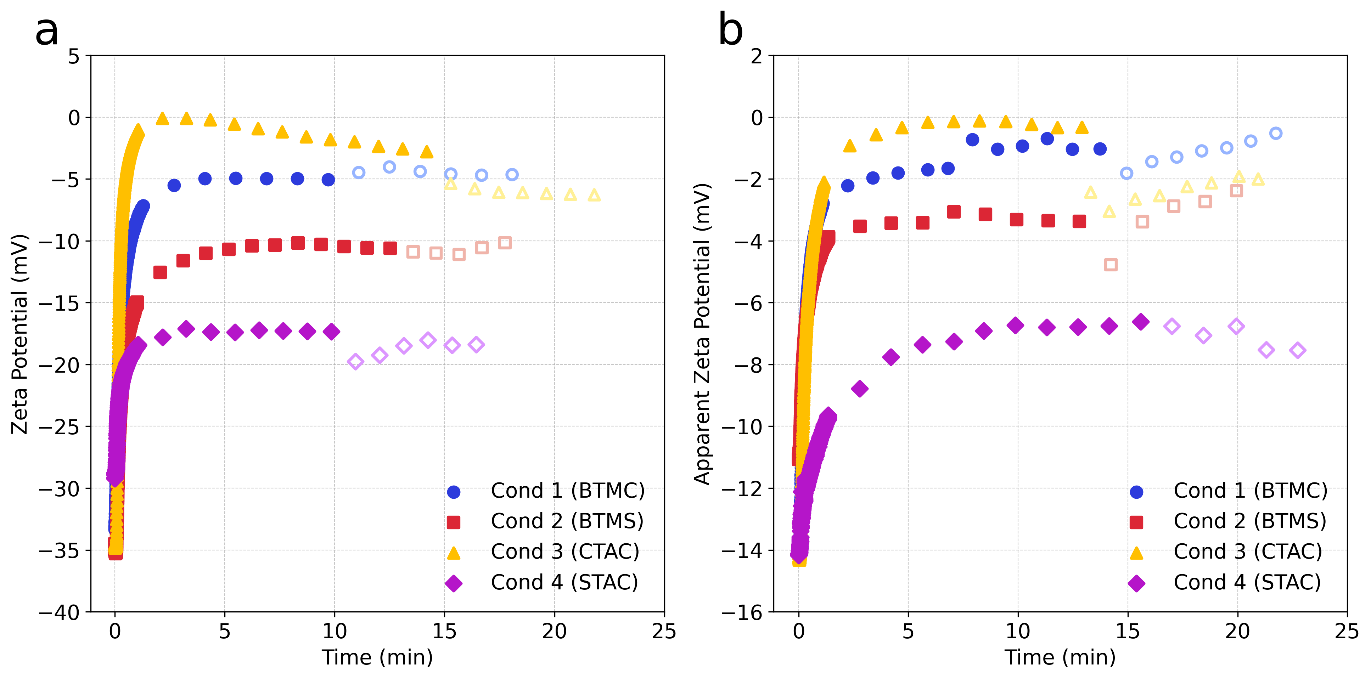


Figure S5: Sorption kinetics of a) healthy hair, and b) damaged hair when the four conditioners (250 ppm) were added into the buffer solution and upon desorption with buffer solution. Filled markers represent adsorption data, while empty markers represent desorption data. Conditioners were added at t = 0 min.

| **Parameter** | **Silicon Oxide Wafer** | **Human Hair** | **Key Similarities/Differences** |
| --- | --- | --- | --- |
| Surface Composition | Homogeneous silanol groups (inorganic) | Heterogeneous (proteins, lipids, fatty acids) | Difference: Wafer lacks organic complexity; hair’s surface varies with damage and biological origin. |
| Surface Morphology | Smooth, flat, homogeneous | Fibrous, micro-roughness | Difference: Wafer’s simplicity enables controlled adsorption studies. |
| Surface Area | Small | Large | Difference: Hair’s irregular surface causes higher surface area |
| ζ-Potential | Highly negative | Less negative | Similarity: Both surfaces are negatively charged. |
| Isoelectric Point (IEP) | IEP ≈3 – 4 | IEP ≈3 – 4 (virgin)  Shifts to lower values with increasing oxidative damage | Similarity: IEP in acidic range |

Table S4: Summary of similarities and differences between Si | SiO_2_ wafer and human hair.


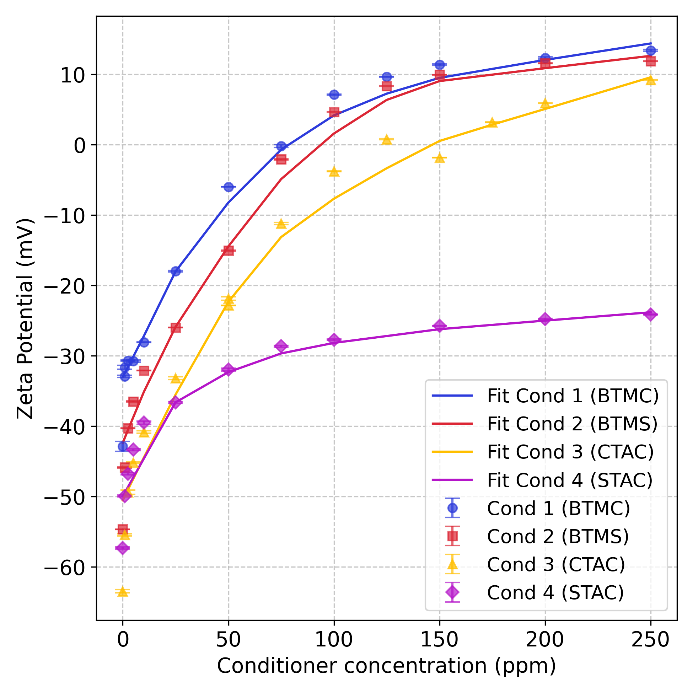


Figure S6: Changes in zeta potential with increasing conditioner concentration added to Si | SiO_2_ wafer.

| IEP values | Si⏐SiO_2_ | Conditioner #1 | Conditioner #2 | Conditioner #3 | Conditioner #4 |
| --- | --- | --- | --- | --- | --- |
| Adsorption | 3.91 | 9.98 | 9.84 | 8.99 | 4.32 |
| Desorption |  | 6.72 | 5.95 | 6.68 | 4.03 |
| Change |  | 3.26 | 3.89 | 2.31 | 0.29 |

Table S5: Summary of IEP values for Si | SiO_2_ wafer with different conditioner treatment.


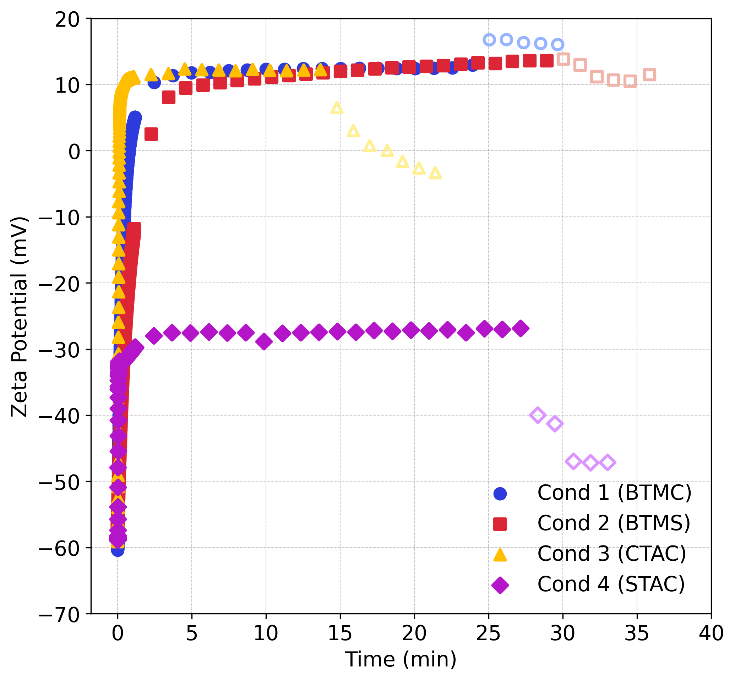


Figure S7: Sorption kinetics of Si | SiO_2_ wafer when Conditioners #1 – #4 (250 ppm) were added into the buffer solution and upon desorption with buffer solution. Filled markers represent adsorption data, while empty markers represent desorption data. Conditioners were added at t = 0 min


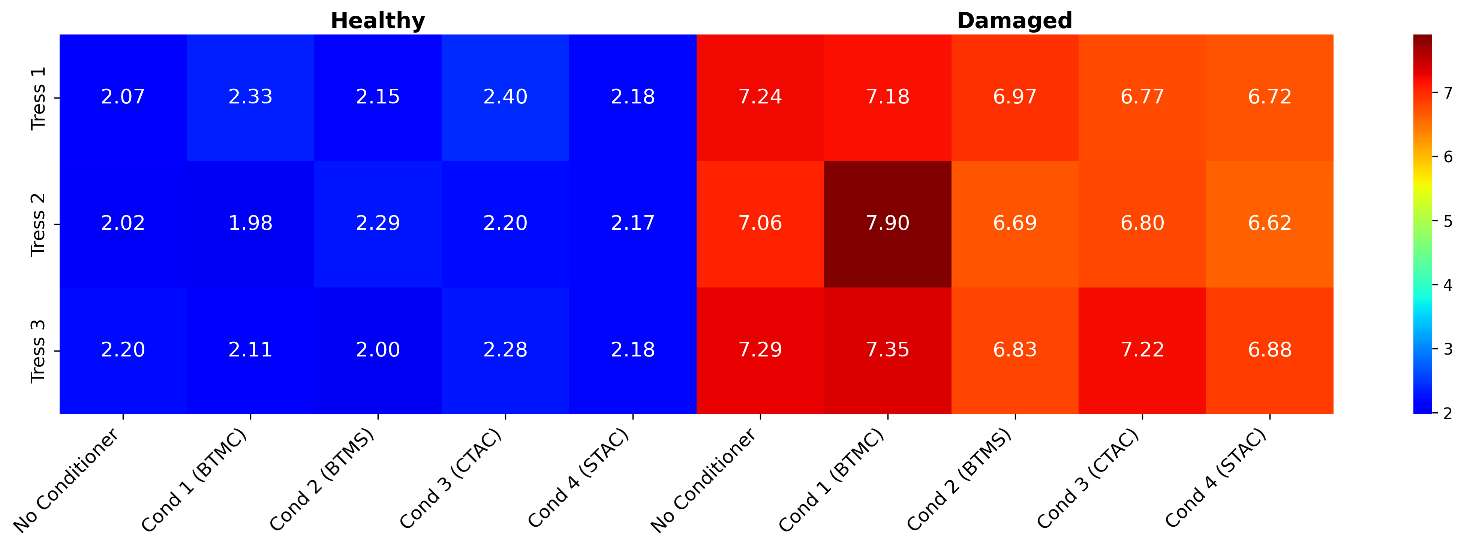


Figure S8. Cysteic acid levels measured by ATR-IR of healthy and damaged hair tresses after application of different conditioners. Values are arbitrary and higher values indicate higher cysteic acid levels.
